# Effects of Valproate and Deferoxamine on Epileptiform Activity, Oxidative Stress, and Nitric Oxide in a Penicillin-Induced Epilepsy Model

**DOI:** 10.3390/biology15161375

**Published:** 2026-08-12

**Authors:** Arzuhan Çetindağ, Şeyma Özsoy, Levent Hacisüleyman, Ziya Çakir, Elif Azize Özşahin Delibaş

**Affiliations:** 1 Vocational School of Health Services, Sivas Cumhuriyet University, 58140 Sivas, Türkiye; acetindag@cumhuriyet.edu.tr; 2 Department of Physiology, Faculty of Medicine, Tokat Gaziosmanpasa University, 60100 Tokat, Türkiye; seyma.ozsoy@hotmail.com; 3 Department of Pharmacology, Faculty of Pharmacy, Fenerbahçe University, 34758 Istanbul, Türkiye; 4 Department of Oral and Dental Health, Faculty of Health Services Vocational School, Tokat Gaziosmanpasa University, 60100 Tokat, Türkiye; cakirziya@yahoo.com; 5 Department of Nutrition and Dietetics, Faculty of Health Sciences, Tokat Gaziosmanpasa University, 60100 Tokat, Türkiye; elif.delibas@gop.edu.tr

**Keywords:** epilepsy, valproate, deferoxamine, oxidative stress, nitric oxide, electrocorticography

## Abstract

Oxidative and nitrosative stress contribute to epileptogenesis and seizure-related neuronal injury through iron-mediated reactive oxygen species generation and excessive nitric oxide production. This study examined whether combining valproate (VALP) with the iron chelator deferoxamine (DFO) produces additional electrophysiological or biochemical effects in a penicillin-induced model of epileptiform activity. Male Wistar rats were assigned to four groups (*n* = 7/group): penicillin control, VALP (500 mg/kg), DFO (100 mg/kg), and VALP + DFO. Epileptiform activity was induced by intracortical penicillin and monitored by electrocorticography for 180 min, and spike frequency, spike amplitude, serum and hippocampal total antioxidant status, total oxidant status, oxidative stress index, and nitric oxide levels were measured. VALP, DFO, and their combination significantly reduced spike frequency and amplitude versus penicillin. Although VALP + DFO did not further suppress spike frequency or spike amplitude compared with VALP alone, the combination was associated with selected biochemical differences, including lower serum and hippocampal nitrite/nitrate surrogate levels and higher hippocampal total antioxidant status. These findings suggest that VALP + DFO produces selected, marker-specific biochemical changes beyond VALP monotherapy, without providing additional anticonvulsant efficacy in this acute model. The functional significance of these biochemical changes remains to be established.

## Simple Summary

Epilepsy is a common neurological disorder in which abnormal electrical activity in the brain causes recurrent seizures. Increasing evidence suggests that oxidative damage and excessive production of nitric oxide, a signaling molecule that can become harmful in large amounts, contribute to brain injury during seizures. This study investigated whether combining the widely used anti-seizure medicine valproate with deferoxamine, a drug that removes excess iron from the body, alters markers of oxidative and nitrosative stress in the brain, in addition to its effects on seizure-related electrical activity. Using an experimental model of epilepsy in rats, we found that both treatments reduced seizure-related electrical activity. Although the combination did not suppress seizures more effectively than valproate alone, it was associated with lower nitrite/nitrate surrogate markers of nitric oxide metabolism and higher hippocampal antioxidant status, while its effects on other oxidative stress markers were mixed. These findings indicate that combining these two agents produces measurable, marker-specific biochemical changes in this acute experimental model. However, the combination did not provide additional suppression of epileptiform activity compared with valproate alone, and whether the biochemical changes have functional significance remains to be determined in future studies.

## 1. Introduction

Epilepsy is a chronic neurological condition defined by the presence of repeated, unprovoked seizures. It affects nearly 50 million individuals across the globe, yet an estimated 40 million remain untreated, with the vast majority—around 85%—residing in low- and middle-income countries [1]. Epileptogenesis is a complex process in which various molecular and cellular disturbances transform normal neural circuits into hyperexcitable networks capable of generating recurrent seizures. Key contributors include neuroinflammation driven by cytokines such as IL-1β and TNF-α, disruption of the blood–brain barrier, dysregulation of signaling pathways like mTOR, and abnormalities in autophagy and apoptosis. Altered ion channel function, excitotoxicity, and hippocampal remodeling further promote neuronal hyperexcitability. Moreover, ongoing oxidative stress together with mitochondrial dysfunction appears to help maintain these pathological changes, while disturbances in thalamocortical circuitry contribute particularly to absence-type seizures [2].

The brain’s heavy reliance on oxidative metabolism causes it to consume large amounts of oxygen and produce considerable reactive oxygen species (ROS). While antioxidant defenses normally maintain redox balance, aging is often accompanied by neurodegenerative changes marked by the buildup of redox-active iron, which further contributes to oxidative imbalance [3]. Iron acts as a potent pro-oxidant, and within the reducing conditions of the intracellular environment, it facilitates hydroxyl radical formation via the Fenton reaction [4].

ROS and reactive nitrogen species (RNS) are constantly formed during normal cellular metabolism. When the generation of these species overwhelms antioxidant defense capacity, an imbalance occurs and oxidative stress develops, resulting in cellular dysfunction and death. Within the central nervous system, oxidative and nitrative stress, together with neuroinflammatory processes, are recognized as key contributors to the pathogenesis of neurological conditions, including epilepsy, stroke, neurodegeneration, and malignancies [5]. The brain is especially vulnerable to oxidative injury because of its high metabolic demand, containing large amounts of polyunsaturated fatty acids, and relatively limited antioxidant capacity. Notably, enzymatic antioxidants, including catalase and glutathione peroxidase are expressed at substantially lower levels in neural tissue compared to peripheral organs, rendering neurons especially susceptible to ROS-mediated damage [6]. Nitric oxide (NO) is another reactive mediator that contributes to seizure-related neuronal injury. During epileptic activity, excessive Ca^2++^ influx activates neuronal nitric oxide synthase (nNOS) and augments NO generation [7]. Elevated NO levels have been associated with seizure-induced neurotoxicity, as evidenced by reports showing that neuronal loss is markedly reduced in nNOS-deficient mice exposed to convulsant stimuli [8]. These findings suggest that NO contributes to excitotoxic and degenerative processes during epileptogenesis.

The brain’s heightened vulnerability is further aggravated when pro-oxidant processes, such as iron accumulation or mitochondrial dysfunction, amplify ROS generation and promote neuronal hyperexcitability. Excess cellular iron is particularly detrimental because it can trigger ferroptosis, a regulated form of cell death involving iron-driven lipid peroxidation and oxidative damage, which further exacerbates neuronal vulnerability and may contribute to seizure development [9]. In light of these mechanisms, multiple studies have reported beneficial effects of iron chelation therapy in epilepsy [10,11,12], suggesting that limiting iron availability may influence oxidative-stress-related biomarkers and seizure-associated injury. This provides a rationale for therapeutic strategies that combine conventional antiepileptic drugs with iron chelators. In this context, sodium valproate (VALP) and deferoxamine (DFO) may exert complementary effects by reducing iron-dependent oxidative stress and NO–mediated neurotoxicity. The present study therefore examined the anticonvulsant effects and the associated changes in oxidative and nitrosative stress markers following VALP, DFO, and their combination in an acute penicillin-induced model of epileptiform activity.

## 2. Materials and Methods

### 2.1. Experimental Animals

In this study, 28 adult male Wistar albino rats weighing 200–250 g and aged 4–5 months were used. Male rats were used to minimize the potential confounding effects of hormonal fluctuations associated with the estrous cycle on seizure susceptibility and oxidative stress parameters, thereby reducing biological variability between experimental groups [13,14]. The animals were obtained from the Tokat Gaziosmanpaşa University Animal Laboratory. They were housed under standard laboratory conditions with a 12:12 h light–dark cycle (lights on from 07:00 to 19:00). Rats had free access to standard laboratory chow and tap water. The study protocol was approved by the Tokat Gaziosmanpaşa University Animal Experiments Local Ethics Committee (HADYEK), approval number 2023-HADYEK-07, dated 5 May 2023.

### 2.2. Chemicals

Penicillin (Sigma-Aldrich, St. Louis, MO, USA), DFO (Desferal^®^ 500 mg vial, Novartis Pharma Stein AG, Stein, Switzerland), and VALP (Sanofi-Aventis Co., Sanofi, France) were used in this study. Penicillin was dissolved in physiological saline prior to administration and injected intracortically (i.c.) through a skull opening. DFO and VALP were administered intraperitoneally (i.p.) 30 min after penicillin injection.

### 2.3. Experimental Design and Treatment Groups

The animals were randomly assigned to four experimental groups (*n* = 7 per group) using a simple randomization procedure with a computer-generated random number sequence prepared by an independent investigator. The sample size was chosen based on our previously published studies using the same penicillin-induced epilepsy model and comparable outcome measures [15,16], which informed the experimental design and feasibility of the present study.

- Group 1 (Penicillin control group): Penicillin (500 IU, 2.5 µL, i.c.) followed by i.p. administration of 1 mL physiological saline.
- Group 2 (VALP group): Penicillin (500 IU, 2.5 µL, i.c.) followed by VALP (500 mg/kg, i.p.).
- Group 3 (DFO group): Penicillin (500 IU, 2.5 µL, i.c.) followed by DFO (100 mg/kg, i.p.).
- Group 4 (VALP + DFO group): Penicillin (500 IU, 2.5 µL, i.c.) followed by combined treatment with DFO (100 mg/kg, i.p.) and VALP (500 mg/kg, i.p.).

VALP and/or DFO were administered i.p. 30 min after penicillin injection in all treatment groups. The dose of sodium valproate (500 mg/kg) was selected based on previous studies demonstrating significant anticonvulsant efficacy in experimental epilepsy models [16]. Similarly, the dose of deferoxamine (100 mg/kg) was chosen according to previous experimental studies reporting effects on seizure-related activity and oxidative-stress markers through attenuation of iron-mediated oxidative stress [11].

### 2.4. Surgical Procedure and Electrocorticographic (ECoG) Recording

All experimental procedures were conducted in the Physiology Laboratory of Gaziosmanpaşa University Faculty of Medicine. Following initial anesthesia with urethane (1.25 g/kg i.p.), the rats were placed in a stereotaxic apparatus and securely immobilized (Harvard Apparatus, Holliston, MA, USA). A rostrocaudal midline incision of (~3 cm) was made in the scalp, and soft tissues overlying the left somatomotor cortex were carefully removed. The skull was gently thinned using a rotary drill to expose the cortex.

ECoG procedures were adapted from Acungil et al. [16]. Epileptiform activity was induced by i.c. microinjection of penicillin G potassium (500 IU in 2.5 μL saline; AP −2 mm, ML +3 mm from bregma, DV −1 mm) using a Hamilton syringe (Hamilton Company, Reno, NV, USA). Two Ag/AgCl ball electrodes were placed over left somatomotor cortex (both 2 mm lateral to midline: positive at 1 mm anterior to bregma; negative at 5 mm posterior to bregma). An Ag/AgCl clip electrode on the left ear served as common reference. Body temperature was kept at 37 °C using a homeothermic heating blanket with rectal probe (Harvard Apparatus, Holliston, MA, USA).

ECoG recordings were initiated before penicillin injection and continued for a total of 210 min. Following penicillin injection, epileptiform activity was allowed to stabilize for 30 min before treatment administration. Baseline spike frequency and spike amplitude were calculated as the mean values obtained from 1-min recording epochs acquired during the final 10 min immediately before treatment administration. Following treatment administration, spike frequency and spike amplitude were analyzed from 1-min recording epochs obtained at 10-min intervals throughout the subsequent 180-min recording period. Treatment drugs were administered 30 min after penicillin injection, and ECoG recordings were continued for an additional 180 min. Cortical activity was amplified (EEG-100C), digitized via MP150 system, and analyzed offline using AcqKnowledge software v3.9.1 (Biopac Systems, Inc., Goleta, CA, USA). Spike frequency/amplitude determined by peak-to-peak measurements. The investigator responsible for ECoG data analysis was blinded to the experimental group allocation throughout the analysis. No animals were excluded from the analysis. All recordings met predefined signal-quality criteria.

### 2.5. Biochemical Analyses

Blood samples were collected into serum tubes, allowed to clot ~30 min at room temperature, and centrifuged at 4000× *g* for 10 min at 4 °C. Hippocampi were homogenized in appropriate buffer, and supernatants collected. All samples were stored at −80 °C in Eppendorf tubes until analysis. The investigator performing the biochemical analyses was blinded to the experimental group allocation throughout the experimental procedures.

#### 2.5.1. Measurement of Total Oxidant Status (TOS) and Total Antioxidant Status (TAS)

TOS and TAS levels were measured in both serum and hippocampal tissue samples using Erel’s automated spectrophotometric methods [17,18]. In serum TAS and TOS values were expressed as mmol Trolox equivalent/L and μmol H_222_ equivalent/L, respectively.

In tissue samples, TAS and TOS values were normalized to protein concentration and expressed as mmol Trolox equivalent/mg protein and μmol H_222_ equivalent/mg protein, respectively.

The oxidative stress index (OSI) was calculated for both serum and hippocampal tissue samples as the ratio of TOS to TAS, using the following formula:

OSI (AU) = [(TOS, μmol H_222_ Eq/L)/(TAS, μmol Trolox Eq/L)] × 100 (serum);

analogous ratio for tissue [19].

#### 2.5.2. Determination of Nitrite/Nitrate Surrogate Markers of NO Metabolism

Nitrite/nitrate surrogate markers of nitric oxide (NO) metabolism in serum and hippocampal tissue samples were quantified using an ELISA kit (MyBioSource, cat. no. MBS2604161; San Diego, CA, USA) in accordance with the manufacturer’s protocol (expressed as μmol/L serum; μmol/g protein tissue). This assay reflects nitrite/nitrate surrogate markers of NO metabolism rather than direct measurement of NO gas or its reactive intermediates. Accordingly, the results are interpreted as nitrite/nitrate-related markers of nitrosative stress. Protein concentrations were measured with the bicinchoninic acid (BCA) Protein Assay Kit (Thermo Scientific Pierce, cat. no. 23225; Rockford, IL, USA).

### 2.6. Statistical Analysis

Statistical analyses were performed using SPSS v26.0 (IBM Corp., Armonk, NY, USA). Normality was assessed using the Shapiro–Wilk test.

Because spike frequency and spike amplitude were measured repeatedly in the same animals over time, the primary longitudinal analysis was performed using a linear mixed-effects model. Group, Time, and the Group × Time interaction were specified as fixed effects, and Animal was included as a random intercept to account for the correlation among repeated measurements within each animal. Time was entered as a continuous variable. This approach was selected because it accounts for within-animal correlation and does not require the sphericity assumption of repeated-measures ANOVA. Models were fitted using maximum likelihood, and the significance of the fixed effects was assessed using likelihood-ratio tests comparing nested models.

To characterize treatment-group differences at specific recording times, secondary cross-sectional comparisons were also performed at each 10-min interval. Because the distributions of spike frequency and spike amplitude at individual time points deviated from normality, these time-specific comparisons were conducted using the Kruskal–Wallis test, followed, when the overall test was significant, by Mann–Whitney U tests with Bonferroni correction for pairwise comparisons. The adjusted significance threshold for these comparisons was α = 0.0083.

The mean spike frequency and mean spike amplitude calculated for each animal across the entire 180-min post-treatment recording period, as well as the biochemical variables, were analyzed using one-way analysis of variance (ANOVA) followed by Tukey’s honestly significant difference (HSD) test when the relevant parametric assumptions were met. The biochemical variables included total antioxidant status (TAS), total oxidant status (TOS), oxidative stress index (OSI), and nitrite/nitrate surrogate markers of nitric oxide metabolism. Effect sizes for the one-way ANOVA results are reported as partial eta squared (partial η^2^).

Data are presented as mean ± standard error of the mean (SEM). Statistical significance was set at *p* < 0.05, except for the Bonferroni-adjusted time-specific pairwise comparisons, for which *p* < 0.0083 was considered statistically significant.

## 3. Results

### 3.1. Spike Frequency

Linear mixed-effects analysis demonstrated significant effects of Group and Time for both spike frequency and amplitude, together with a significant Group × Time interaction (all *p* < 0.0001), indicating that changes over time differed by treatment group and were not attributable to a uniform between-group shift. Specifically, the Penicillin group maintained higher spike frequency and amplitude throughout the recording period, whereas the VALP, DFO, and VALP + DFO groups showed declines over time. However, the VALP + DFO combination did not significantly suppress spike frequency or spike amplitude beyond the effect observed with VALP monotherapy (Table 1).

**Figure 1:**
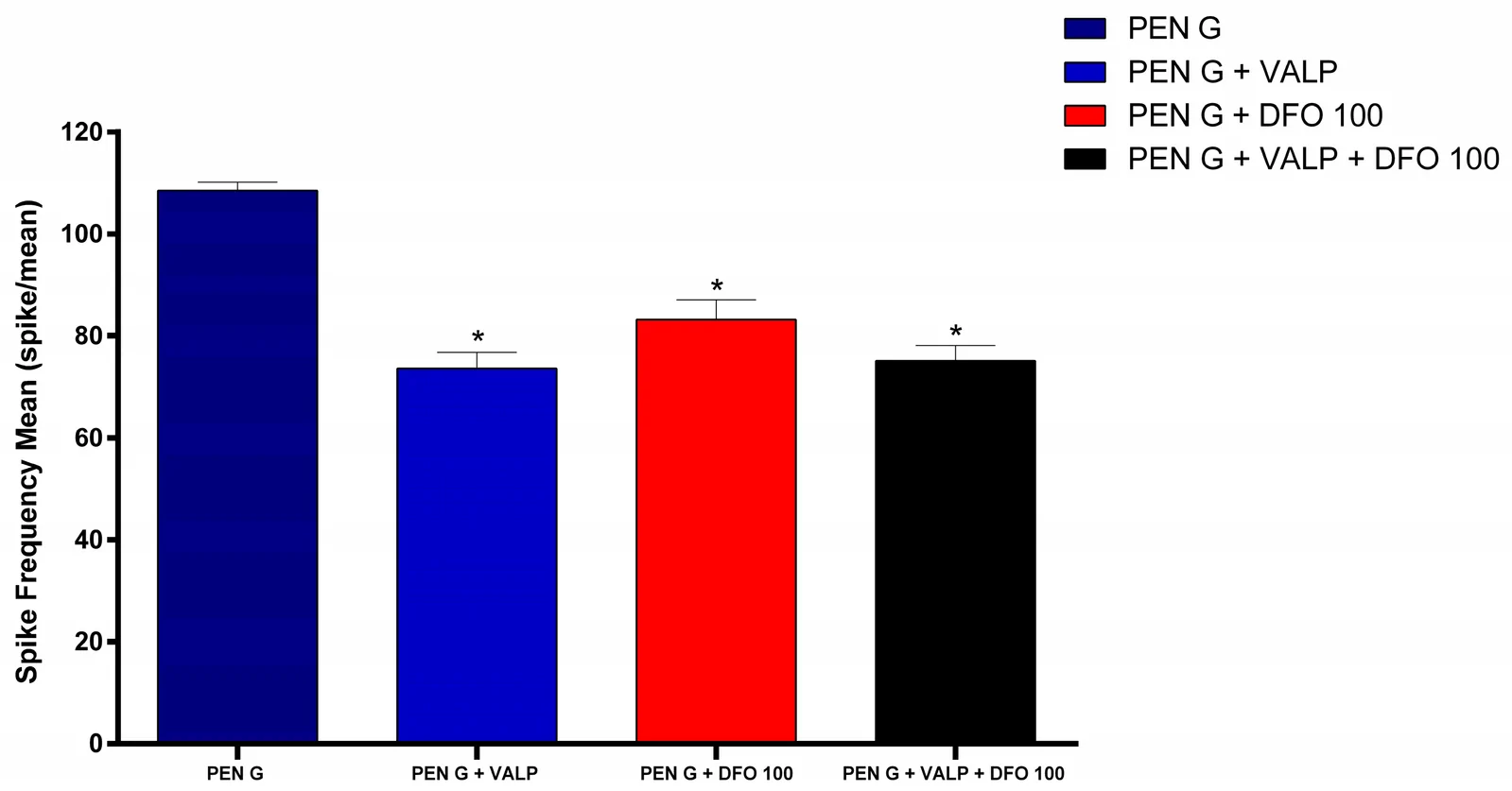
Mean spike frequency (spikes/min) of ECoG activity across experimental groups. Vertical bars represent mean ± SEM. * *p* < 0.001; compared with the penicillin group.

Mean spike frequency stabilized at 108.5 ± 1.63 spikes/min during the baseline period (20–30 min post-penicillin). Administration of VALP, DFO, or their combination significantly reduced spike frequency over the 180-min recording period compared with the penicillin control group (Kruskal–Wallis test, *p* < 0.05; Figure 1). Mean spike frequencies were 73.57 ± 3.23 spikes/min in the penicillin + VALP group, 83.21 ± 3.78 spikes/min in the penicillin + DFO group, and 74.15 ± 2.94 spikes/min in the penicillin + VALP + DFO group (Figure 1; Table 2).

Mean spike frequency differed significantly among the experimental groups (F = 11.79, *p* < 0.001, partial η^2^ = 0.596), indicating a large treatment effect. The mean spike value of the penicillin group was significantly higher than all other groups (Tukey HSD: PEN vs. VALP, mean difference = 34.99, 95% CI 16.65–53.33, *p* < 0.001; PEN vs. DFO, mean difference = 25.33, 95% CI 6.99–43.67, *p* = 0.004; PEN vs. VALP + DFO, mean difference = 33.19, 95% CI 14.85–51.53, *p* < 0.001). VALP, DFO, and VALP + DFO groups exhibited significantly lower mean spike frequencies than the penicillin group. However, no statistically significant differences were observed among the treatment groups (VALP vs. DFO, *p* = 0.480; VALP vs. VALP + DFO, *p* = 0.993; DFO vs. VALP + DFO, *p* = 0.643) (Figure 1).

**Figure 2:**
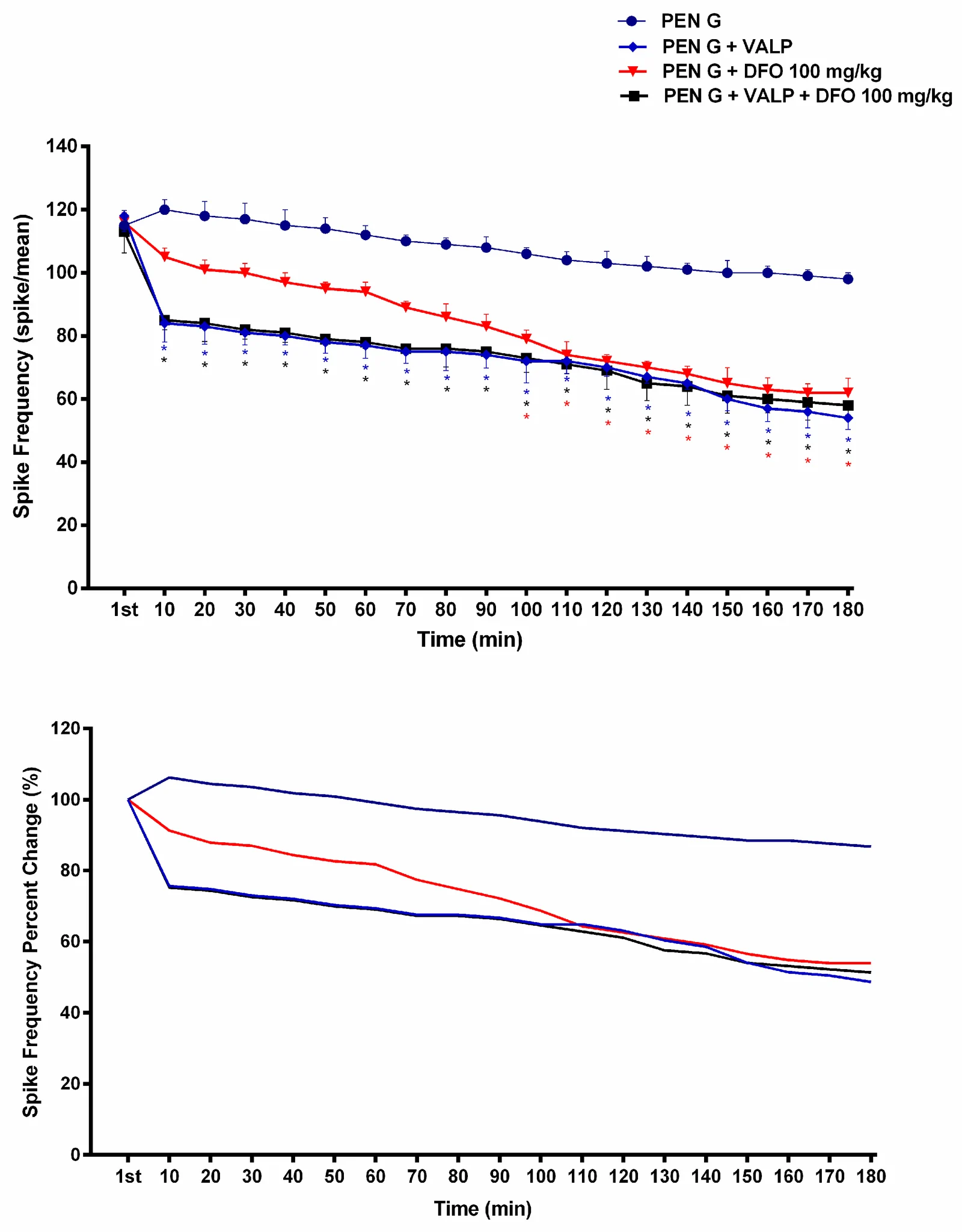
Changes in spike frequency and percentage change in spike frequency over time across experimental groups. The upper graph shows mean spike frequency (spikes/min) values obtained from ECoG recordings following penicillin microinjection. The lower graph shows the percentage change in spike frequency relative to baseline values. * *p* < 0.0083 (Bonferroni-adjusted); significantly different from the penicillin group at the corresponding time point (Mann–Whitney U test following significant Kruskal–Wallis test). Colored lines identify the experimental groups. Colored asterisks indicate a significant difference from the Penicillin group at the corresponding time point; blue asterisks indicate the VALP group, red asterisks indicate the DFO group, and black asterisks indicate the VALP + DFO group.

Baseline spike frequencies were comparable among groups (approximately 115–118 spikes/min). From the 10th minute onward, all treatment groups exhibited significantly lower spike frequencies compared with the penicillin group (*p* < 0.05, Mann–Whitney U test), whereas no significant reduction over time was observed in the penicillin group. All treatment groups showed significant decreases relative to their own baseline values, with the effect of DFO becoming statistically significant after the 100th minute (*p* < 0.05) (Figure 2).

Percentage reductions in spike frequency over 180 min are also shown in Figure 2. VALP, DFO, and VALP + DFO treatments reduced spike frequency by approximately 46%, 38%, and 43%, respectively, all of which were significantly greater than the penicillin group (*p* < 0.05).

### 3.2. Spike Amplitude

**Figure 3:**
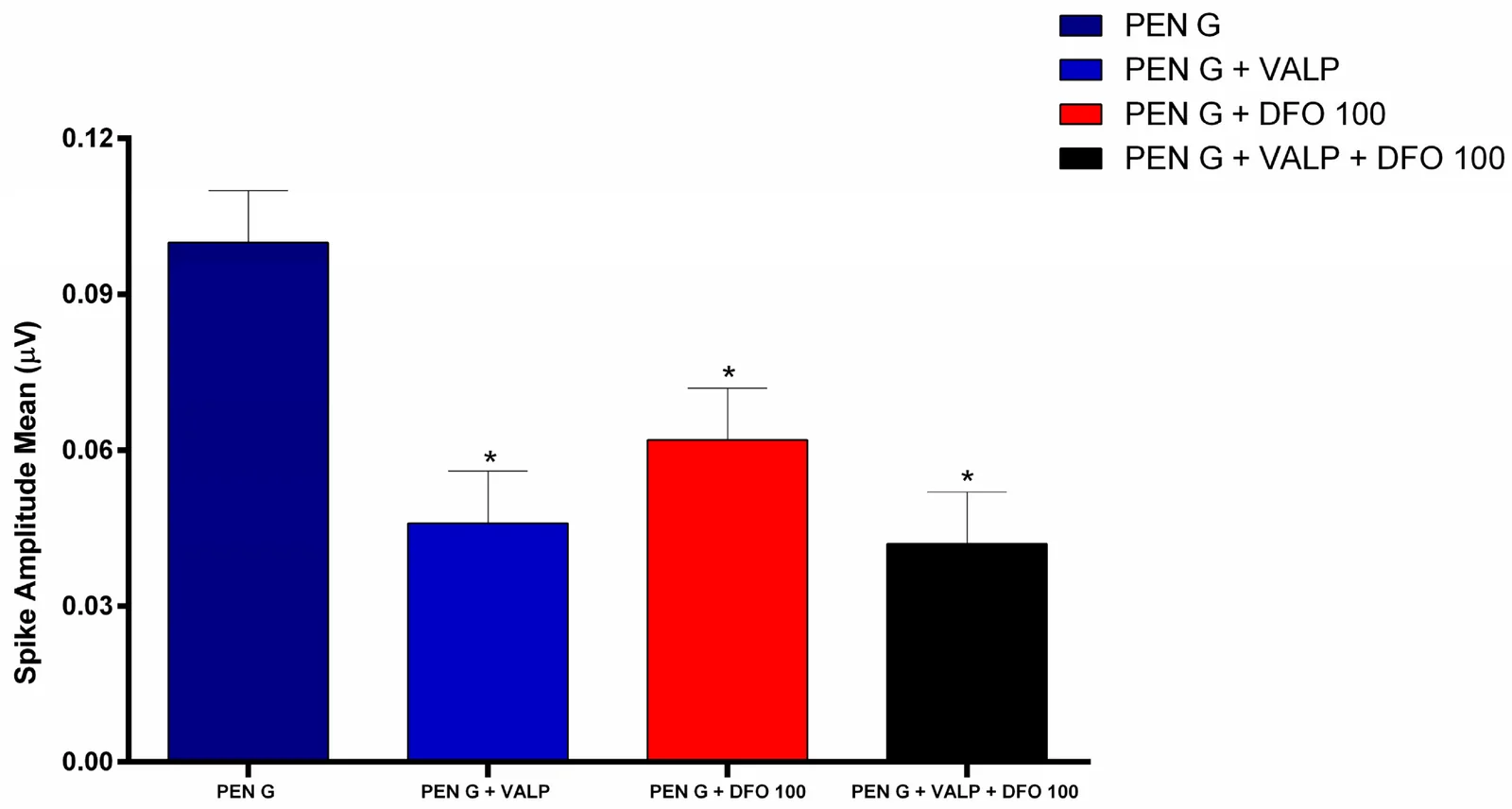
Mean spike amplitude (µV) of ECoG activity across experimental groups. Vertical bars represent mean ± SEM. * *p* < 0.001; compared with the penicillin group.

Mean spike amplitude values differed significantly among groups (F = 29.98, *p* < 0.001, partial η^2^ = 0.789) (Figure 3). The penicillin group exhibited the highest mean spike amplitude (0.100 ± 0.010 μV), which was significantly greater than those observed in the VALP (0.049 ± 0.010 μV), DFO (0.062 ± 0.010 μV), and VALP + DFO (0.042 ± 0.010 μV) groups (Tukey HSD, all *p* < 0.001; 95% CI for mean differences: 0.037–0.076, 0.021–0.061, and 0.041–0.080, respectively). No statistically significant differences were observed among the VALP, DFO, and VALP + DFO groups (Tukey HSD, *p* = 0.162 for VALP vs. DFO; *p* = 0.943 for VALP vs. VALP + DFO; *p* = 0.054 for DFO vs. VALP + DFO).

**Figure 4:**
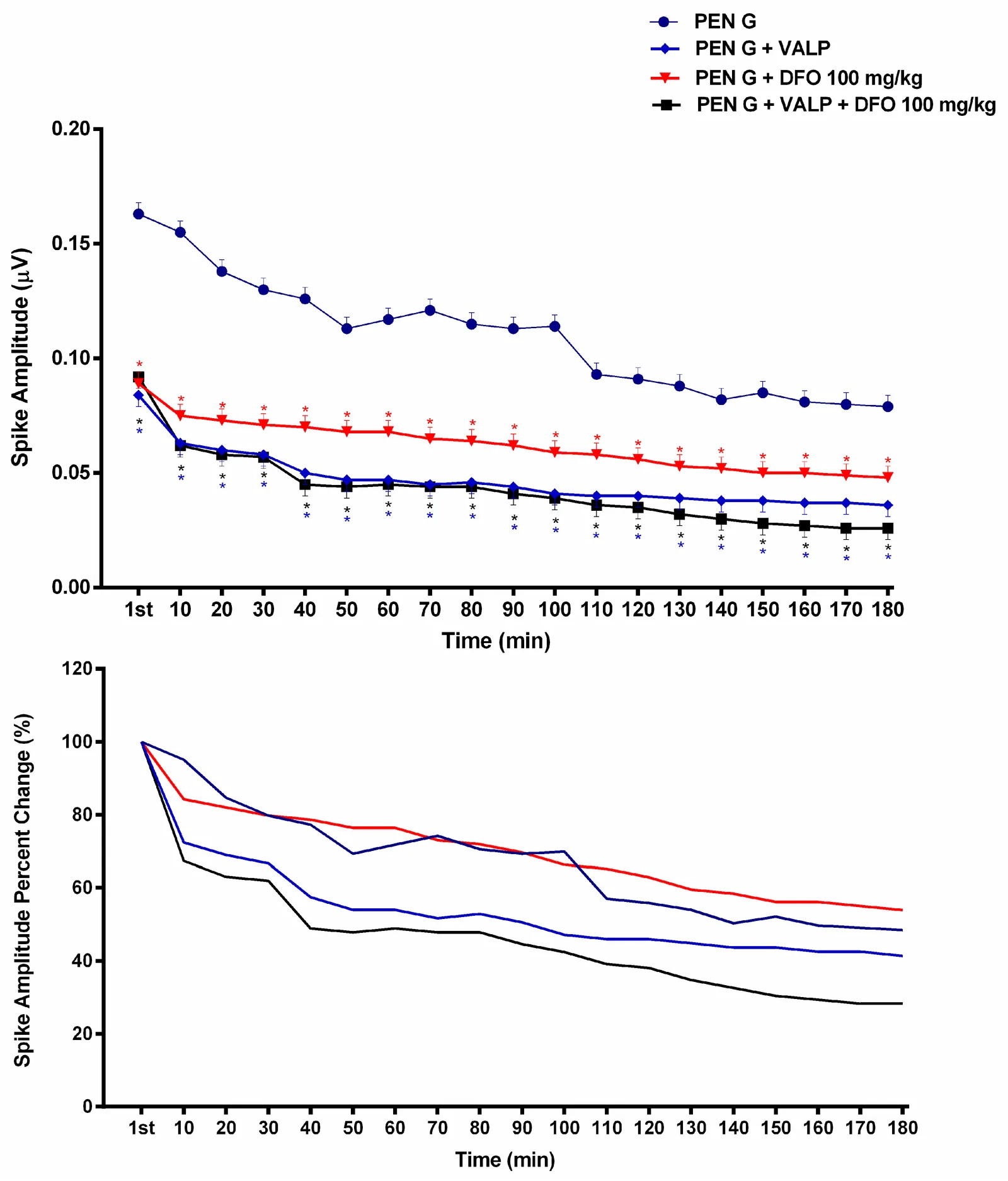
Mean spike amplitude (µV) values and percentage change in spike amplitude across experimental groups. The top graph shows mean spike amplitude (µV) values obtained from ECoG recordings following penicillin injection. The bottom graph shows the percentage change in spike amplitude over the 180-min recording period. * *p* < 0.0083 (Bonferroni-adjusted); significantly different from the penicillin group at the corresponding time point (Mann–Whitney U test following significant Kruskal–Wallis test). Colored lines identify the experimental groups. Colored asterisks indicate a significant difference from the Penicillin group at the corresponding time point; blue asterisks indicate the VALP group, red asterisks indicate the DFO group, and black asterisks indicate the VALP + DFO group.

Pre-treatment baseline spike amplitudes differed significantly among groups, with lower baseline values in the VALP, DFO, and VALP + DFO groups than in the Penicillin group. Accordingly, percentage change from baseline was used only for descriptive visualization, whereas formal inference for the longitudinal data was based on the repeated-measures mixed-effects model. Temporal changes in spike amplitude over the 180-min recording period are shown in Figure 4. At all-time points, spike amplitudes in the VALP, DFO, and VALP + DFO groups were significantly lower than those in the penicillin group (*p* < 0.05). Although the VALP + DFO group consistently exhibited lower spike amplitudes than the VALP group alone, this difference did not reach statistical significance (*p* > 0.05). Spike amplitudes in the penicillin group decreased significantly over time relative to baseline (*p* < 0.05), and all groups showed a significant reduction in spike amplitude within the first 10 min compared with baseline values (*p* < 0.05).

Percentage changes in spike amplitude relative to baseline are also presented in Figure 4. At 180 min, spike amplitude decreased by approximately 52% in the penicillin group, 59% in the VALP group, 44% in the DFO group, and 70% in the VALP + DFO group. Mean spike amplitude values measured over the 180-min recording period, as well as baseline values for each group, are summarized in Table 3. Spike amplitudes ranged from 0.084 to 0.092 μV in the treatment groups and were significantly lower than those of the penicillin group at all time points (*p* < 0.05). Notably, spike amplitude values in the VALP, DFO, and VALP + DFO groups were already significantly lower than the penicillin group at 1 min post-treatment, a time point too early for a genuine pharmacodynamic anticonvulsant effect of intraperitoneally administered VALP or DFO. This early difference more plausibly reflects pre-existing inter-animal variability in penicillin-induced epileptiform severity at the time of randomization, transient effects related to handling or injection stress common to all groups, or other procedural factors unrelated to drug pharmacodynamics, rather than a true baseline physiological state. To account for this variability, treatment effects were additionally evaluated as percentage change relative to each group’s own pre-treatment value (Figure 4), allowing interpretation of drug effects independent of these early between-group differences.

### 3.3. Serum Oxidative Stress Markers (TAS, TOS, OSI) in Experimental Groups

**Figure 5:**
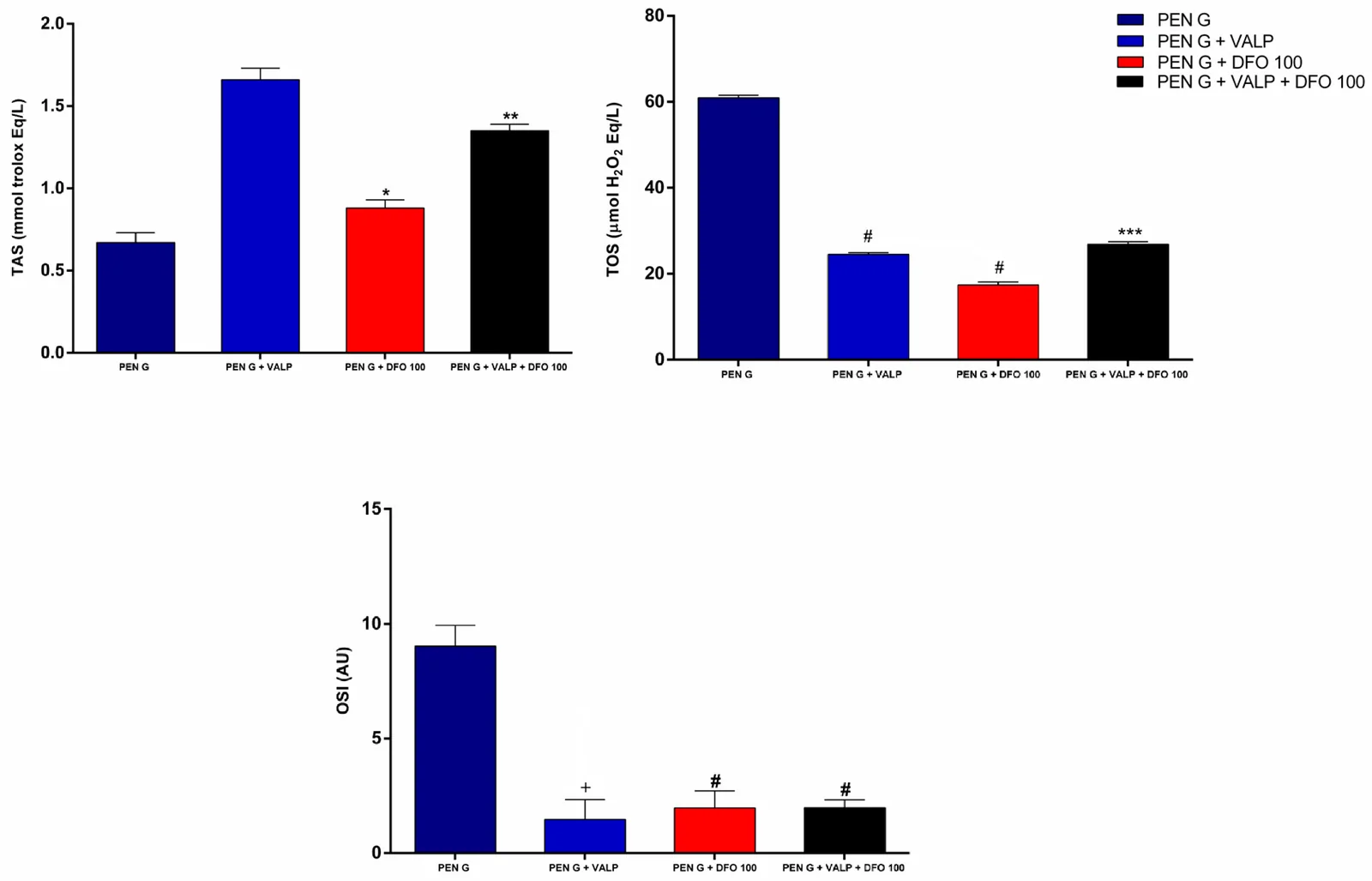
Serum TAS, TOS, and OSI levels in the experimental groups. * *p* < 0.05; different compared to Penicillin, VALP and VALP + DFO groups. ** *p* < 0.01; different compared to Penicillin, VALP and DFO groups. *** *p* < 0.01; different compared to Penicillin and DFO groups. ^+^ *p* < 0.05; different compared to Penicillin, DFO and VALP + DFO groups. ^#^ *p* < 0.001; different compared to Penicillin group.

Serum TAS, TOS, and OSI levels are summarized in Table 4 and illustrated in Figure 5.

Serum TAS levels differed significantly among the experimental groups (F = 88.51, *p* < 0.001, partial η^2^ = 0.917). The lowest TAS level was observed in the penicillin group (0.67 ± 0.06 mmol Trolox Eq/L), indicating a marked reduction in systemic antioxidant capacity. VALP treatment resulted in the highest TAS level (1.66 ± 0.07 mmol Trolox Eq/L). The DFO group exhibited a significantly lower TAS level than the VALP and VALP + DFO groups but remained significantly higher than the penicillin group (*p* < 0.05). Combination treatment (VALP + DFO) significantly increased TAS compared with the penicillin and DFO groups (both *p* < 0.001) but did not reach the level observed with VALP monotherapy (mean difference = 0.312, 95% CI 0.126–0.499, *p* = 0.001).

Serum TOS levels also differed significantly among groups (F = 114.01, *p* < 0.001, partial η^2^ = 0.934) and were significantly higher in the penicillin group (60.96 ± 0.60 µmol _22_O_2_ Eq/L) than in all treatment groups (*p* < 0.001). The DFO-treated group exhibited the lowest serum TOS level (17.40 ± 0.70 µmo_22_H_2_O_2_ Eq/L). VALP (24.50 ± 0.40 µ_22_L H_2_O_2_ Eq/L) showed a borderline non-significant difference from DFO (mean difference = 7.10, 95% CI −0.004 to 14.20, *p* = 0.050), whereas VALP + DFO (26.86 ± 0.60_22_mol H_2_O_2_ Eq/L) was significantly higher than DFO (mean difference = 9.46, 95% CI 2.35–16.56, *p* = 0.006). No significant difference was observed between VALP and VALP + DFO (*p* = 0.797).

Consistent with TOS findings, the highest OSI value was detected in the penicillin group (9.02 ± 0.8 AU), indicating a pronounced oxidative stress state (F = 1194.8, *p* < 0.001, partial η^2^ = 0.993). OSI values were significantly reduced in the VALP, DFO, and VALP + DFO groups compared with the penicillin group (all *p* < 0.001). Notably, VALP monotherapy showed a significantly lower OSI than both DFO (mean difference = −0.503, 95% CI −0.919 to −0.087, *p* = 0.014) and VALP + DFO (mean difference = −0.520, 95% CI −0.936 to −0.104, *p* = 0.011), while no significant difference was observed between DFO and VALP + DFO (*p* = 0.999).

### 3.4. Hippocampal Oxidative Stress Markers (TAS, TOS, OSI) in Experimental Groups

**Figure 6:**
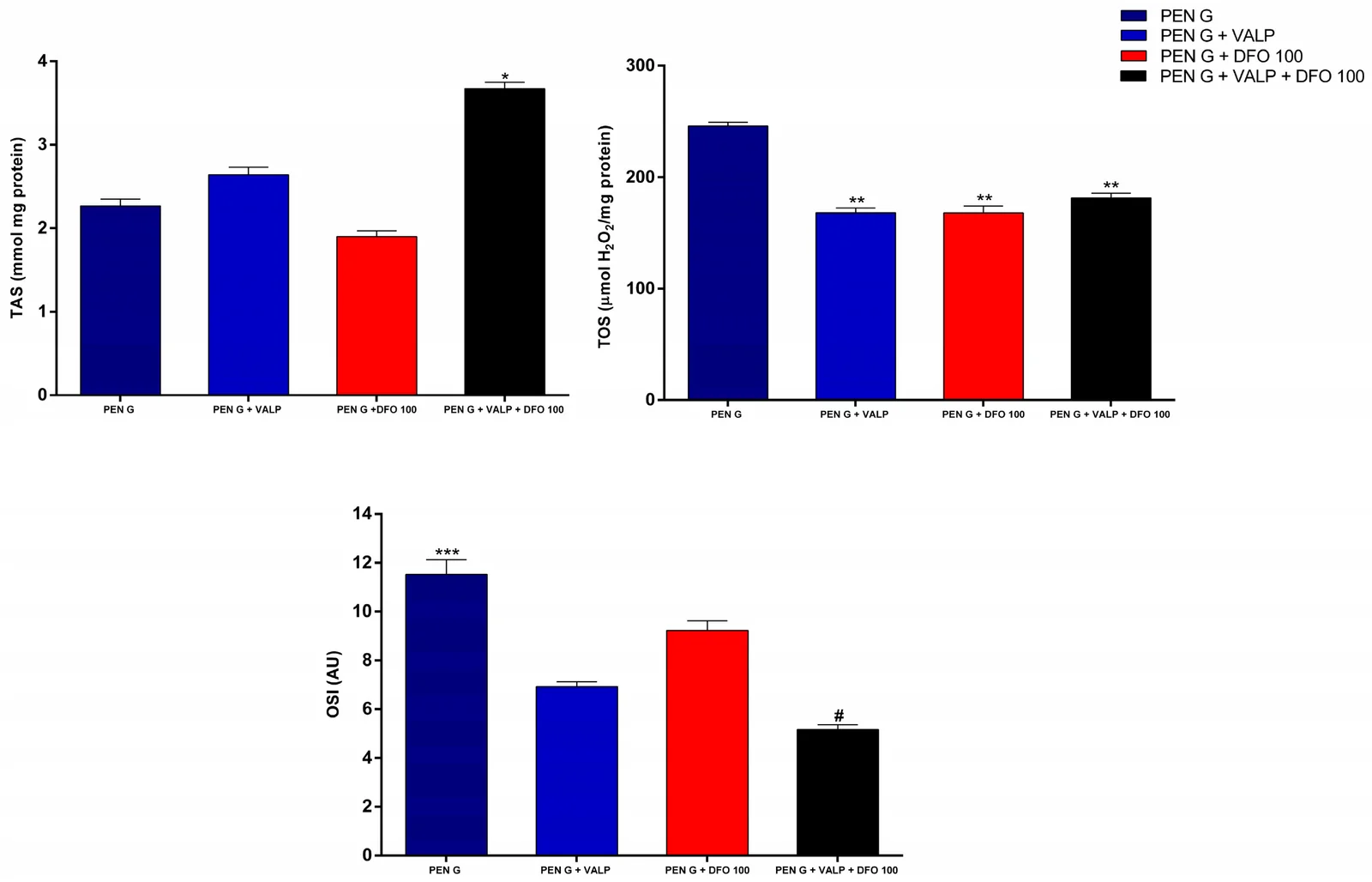
Hippocampal Tissue TAS, TOS, and OSI Levels Across Groups. * *p* < 0.01; significantly different from the Penicillin and DFO groups. ** *p* < 0.001; significantly different from the Penicillin group. *** *p* < 0.01; significantly different from the VALP and VALP + DFO groups. ^#^ *p* < 0.05; significantly different from the Penicillin and DFO groups.

Hippocampal tissue TAS, TOS, and OSI are presented in Table 5 and Figure 6.

Hippocampal TAS levels differed significantly among groups (F = 9.19, *p* < 0.001, partial η^2^ = 0.535). The VALP + DFO combination group showed the highest TAS level (3.67 ± 0.08 mmol Trolox/mg protein), which was significantly higher than the penicillin group (mean difference = 1.41, 95% CI: 0.42 to 2.39, *p* = 0.003), the DFO group (mean difference = 1.77, 95% CI: 0.79 to 2.76, *p* < 0.001), and the VALP group (mean difference = 1.03, 95% CI: 0.05 to 2.01, *p* = 0.038). No significant differences in TAS levels were observed among the penicillin, VALP, and DFO groups (all *p* > 0.05).

Hippocampal TOS levels also differed significantly among groups (F = 18.57, *p* < 0.001, partial η^2^ = 0.699) and were highest in the penicillin group (245.80 ± 3.5 µmol _22_O_2_/mg protein), significantly elevated compared with the VALP group (mean difference = 77.59, 95% CI: 43.94 to 111.25, *p* < 0.001), the DFO group (mean difference = 77.86, 95% CI: 44.21 to 111.52, *p* < 0.001), and the VALP + DFO group (mean difference = 64.33, 95% CI: 30.68 to 97.99, *p* < 0.001), indicating pronounced hippocampal tissue oxidative stress following penicillin administration. No significant differences were observed among the VALP, DFO, and VALP + DFO groups (all *p* > 0.05).

Consistent with these findings, hippocampal OSI values differed significantly among groups (F = 10.40, *p* < 0.001, partial η^2^ = 0.565). The highest OSI value was detected in the penicillin group (11.52 ± 0.6 AU), significantly greater than the VALP group (mean difference = 4.60, 95% CI: 1.25 to 7.95, *p* = 0.005) and the VALP + DFO group (mean difference = 6.37, 95% CI: 3.02 to 9.72, *p* < 0.001), but not significantly different from the DFO group (*p* = 0.258). The lowest OSI level was observed in the VALP + DFO combination group (5.16 ± 0.57 AU), which was significantly lower than the penicillin group and the DFO group (mean difference = −4.07, 95% CI: −7.42 to −0.72, *p* = 0.013), but not significantly different from the VALP group (*p* = 0.478).

### 3.5. Nitrite/Nitrate Surrogate Markers of NO Metabolism in Serum and Hippocampal Tissue

**Figure 7:**
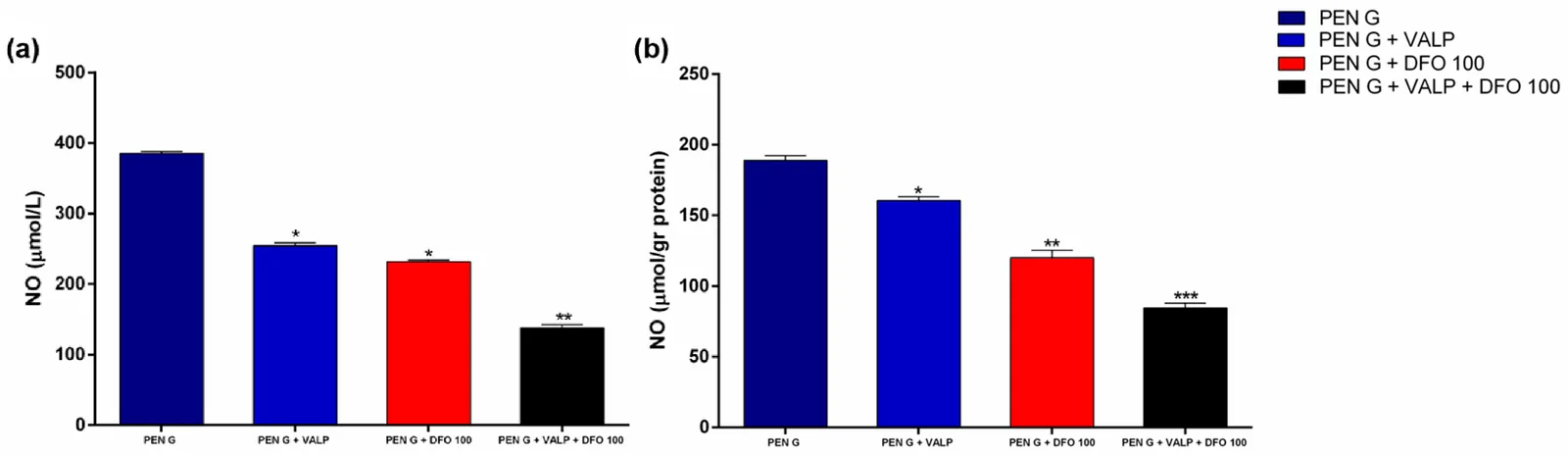
Serum and hippocampal nitrite/nitrate surrogate markers of nitric oxide (NO) metabolism in the experimental groups. (**a**) Serum nitrite/nitrate surrogate marker concentrations across groups. * *p* < 0.001, different compared with Penicillin and VALP + DFO groups. ** *p* < 0.001, different compared with Penicillin, VALP, and DFO groups. (**b**) Hippocampal nitrite/nitrate surrogate marker concentrations across groups.* *p* < 0.001, different compared with Penicillin, DFO and VALP + DFO groups. ** *p* < 0.001, different compared with Penicillin, VALP, and VALP + DFO groups. *** *p* < 0.001, different compared with Penicillin, VALP, and DFO groups.

Serum and hippocampal nitrite/nitrate surrogate marker concentrations, reflecting NO metabolism, are presented in Figure 7.

Serum nitrite/nitrate surrogate marker concentrations differed significantly among groups (F = 71.0, *p* < 0.001, partial η^2^ = 0.899) and were highest in the Penicillin group (385.11 ± 20.53 µmol/L). Concentrations were significantly lower in all treatment groups (Tukey’s HSD, all *p* < 0.001). The VALP (254.51 ± 5.97 µmol/L) and DFO (231.57 ± 9.62 µmol/L) groups had significantly lower concentrations than the Penicillin group (mean difference = 130.60, 95% CI 83.43–177.77, *p* < 0.001; and mean difference = 153.54, 95% CI 106.37–200.71, *p* < 0.001, respectively), but did not differ significantly from each other (mean difference = 22.94, 95% CI −24.23 to 70.11, *p* = 0.546). Both VALP and DFO groups had significantly higher concentrations than the VALP + DFO group (mean difference = 116.51, 95% CI 69.35–163.68, *p* < 0.001; and mean difference = 93.57, 95% CI 46.40–140.74, *p* < 0.001, respectively). The lowest serum nitrite/nitrate surrogate marker concentration was observed in the VALP + DFO group (138.00 ± 5.90 µmol/L), which was significantly lower than that in all other groups (all *p* < 0.001).

Similarly, hippocampal nitrite/nitrate surrogate marker concentrations differed significantly among groups (F = 213.1, *p* < 0.001, partial η2 = 0.964). The highest concentration was observed in the Penicillin group (188.80 ± 2.02 µmol/g protein). The VALP group showed a significantly lower concentration (160.00 ± 2.97 µmol/g protein) than the Penicillin group (mean difference = 28.80, 95% CI 16.62–40.98, *p* < 0.001). A further reduction was observed in the DFO group (120.34 ± 3.89 µmol/g protein), which was significantly lower than the Penicillin group (mean difference = 68.46, 95% CI 56.28–80.63, *p* < 0.001) and the VALP group (mean difference = 39.66, 95% CI 27.48–51.83, *p* < 0.001), but significantly higher than the VALP + DFO group (mean difference = 35.77, 95% CI 23.59–47.95, *p* < 0.001). The lowest hippocampal nitrite/nitrate surrogate marker concentration was detected in the VALP + DFO group (84.57 ± 3.31 µmol/g protein), which was significantly lower than those in the Penicillin, VALP, and DFO groups (all *p* < 0.001).

Because the assay quantified nitrite/nitrate surrogate markers rather than direct gaseous NO, these findings are interpreted as differences in NO-related nitrosative-stress markers and not as direct evidence of altered NO production or nitric oxide synthase activity.

## 4. Discussion

In this study, we evaluated the anticonvulsant effects and associated changes in oxidative and nitrosative stress markers of VALP, DFO, and their combination in an acute penicillin-induced model of epileptiform activity. Penicillin administration produced marked epileptiform activity and was associated with higher nitrite/nitrate surrogate marker concentrations and oxidative-stress indices than those observed in the treatment groups. Both VALP and DFO reduced spike frequency and amplitude and improved markers of oxidative and nitrosative stress, while combined treatment suppressed electrocorticographic activity to a degree comparable to, but not exceeding, VALP alone. Importantly, VALP + DFO did not outperform VALP monotherapy with respect to its principal anticonvulsant effect, indicating that DFO provided no additional electrophysiological benefit over VALP alone in this acute model. Nevertheless, the combination was associated with the most favorable NO profile in both serum and hippocampal tissue and with the highest hippocampal antioxidant capacity. These findings suggest that the two agents may exert complementary biochemical effects rather than a synergistic anticonvulsant interaction. These biochemical differences should be regarded as secondary, exploratory observations in this acute model rather than evidence of an overall therapeutic advantage, and any suggestion that iron chelation broadens the neuroprotective profile of antiepileptic therapy remains a hypothesis warranting further investigation.

Oxidative stress critically contributes to the pathological mechanisms underlying epileptic seizures [20]. Because the brain consumes substantial amounts of oxygen but possesses relatively low antioxidant capacity, it is highly susceptible to oxidative damage [21]. Iron metabolism plays an important role in this process, as free iron ions, especially Fe^2++^, promote the generation of ROS via the Fenton reaction, leading to damage of DNA, lipids, and proteins. Notably, seizure activity itself can promote neuronal iron accumulation, and this iron-ROS interplay has been directly implicated in neuronal dysfunction and pyknotic neuronal injury in both human temporal lobe epilepsy tissue and experimental status epilepticus models, suggesting a feed-forward cycle between seizure activity and iron-mediated oxidative damage [22,23].

Experimental studies have shown that i.c. administration of ferric chloride in rats results in lipid peroxidation and glutathione depletion, whereas treatment with DFO significantly lowers malondialdehyde and restores glutathione, thereby mitigating oxidative stress in the epileptic brain [10]. Consistent with these findings, DFO has been shown to reduce seizure stage based on Racine’s Convulsion Scale, suppress epileptic activity on EEG recordings, and significantly increase superoxide dismutase levels in rats with iron-induced focal epilepsy [11]. In line with these observations, deferoxamine also attenuated seizure severity, oxidative stress, and apoptosis in a pentylenetetrazole induced acute seizure model in rats, supporting its potential effects on seizure-related and oxidative-stress-related outcomes, which require further investigation [12]. These previous findings suggest that DFO may influence seizure-related activity and oxidative-stress-related biomarkers, likely through attenuation of iron-mediated oxidative damage and reinforcement of endogenous antioxidant defenses.

NO has also been implicated in epileptogenesis due to its ability to modulate neuronal excitability, neurotransmission, neuroinflammation, and oxidative/nitrosative stress. Excessive production of NO, particularly through nNOS, can promote seizure generation by enhancing glutamatergic neurotransmission, disrupting mitochondrial function, and increasing neuronal vulnerability to excitotoxic damage. Experimental data in the PTZ model have demonstrated that pharmacological agents that reduce brain NO levels or suppress nNOS expression attenuate seizure severity and delay seizure onset [24,25]. Consistent with these observations, both DFO and VALP were associated with significantly lower hippocampal nitrite/nitrate surrogate marker concentrations than the Penicillin group, with the VALP + DFO group showing the lowest concentrations. These findings suggest that the treatments may alter NO-related nitrosative-stress markers in this model; however, the functional significance of these changes remains uncertain. The ELISA assay measured nitrite/nitrate surrogate markers of NO metabolism rather than direct gaseous NO or its reactive intermediates. Therefore, the present results should not be interpreted as direct evidence of altered NO production or nitric oxide synthase activity.

Previous studies have reported antioxidant and seizure-related biochemical effects of VALP in experimental and clinical epilepsy settings. Clinical studies in pediatric patients have shown that VALP monotherapy enhances systemic antioxidant defenses through increased activities of enzymes such as superoxide dismutase, catalase, and glutathione reductase, while reducing oxidative biomarkers including hydrogen peroxide and malondialdehyde [26]. Consistent with these findings, another clinical study in children with idiopathic epilepsy reported that although VALP treatment was associated with a modest increase in lipid peroxidation compared to untreated patients, oxidative markers remained comparable to healthy controls, and superoxide dismutase activity showed a positive correlation with treatment duration, suggesting adaptive upregulation of antioxidant defenses rather than pathological oxidative stress [27]. However, reports of oxidative stress with VALP are often confined to subgroups developing obesity (e.g., elevated malondialdehyde MDA/low vitamin E after 1 year only in overweight children) [28]. Experimental data in neuronal cell models similarly demonstrate that VALP attenuates oxidative injury and improves cell viability under glutamate-induced excitotoxic conditions [29]. More recent work in a kainic acid hippocampal seizure model shows that VALP suppresses ferroptosis via downregulation of lysyl oxidase, reducing lipid peroxidation and Ptgs2 expression in a manner correlated with anti-seizure efficacy [30]. Collectively, these findings suggest that VALP may modulate seizure-related oxidative stress.

In the present study, co-administration of VALP and DFO produced electrophysiological effects comparable to VALP alone and did not provide additional suppression of spike frequency or amplitude. The combination was nevertheless associated with selected biochemical differences, particularly lower nitrite/nitrate surrogate levels and higher hippocampal TAS, whereas serum oxidative-stress indices did not show a uniform advantage over monotherapy. Although DFO is not primarily recognized as a direct modulator of GABAergic transmission—the primary mechanism underlying penicillin-induced epileptiform activity—it may exert anticonvulsant effects by reducing iron-dependent ROS formation, improving mitochondrial redox balance, and suppressing oxidative and nitrosative pathways that facilitate seizure propagation. These mechanisms represent potential explanations for the anticonvulsant activity observed with DFO monotherapy and warrant direct experimental confirmation, since iron homeostasis, mitochondrial function, and nitric oxide synthase activity were not directly measured in the present study. However, because VALP already provides robust seizure suppression through direct enhancement of GABAergic inhibition, the addition of DFO did not yield further electrophysiological benefit. Even so, the combined biochemical changes may warrant further investigation in chronic epilepsy models or conditions characterized by heightened oxidative burden.

Prior work indicates that VALP can increase the labile iron pool and promote iron-dependent lipid peroxidation in hepatic and cancer cell models, with iron chelators such as DFO reversing these changes and attenuating VALP-induced liver steatosis [12,31,32]. Based on this literature, we propose only as a hypothesis to be tested in future studies, that iron chelation may similarly mitigate VALP-associated ferroptotic mechanisms in the context of long-term antiepileptic therapy; however, no ferroptosis-specific biomarkers (e.g., GPX4, ACSL4, SLC7A11, lipid peroxidation products) or iron homeostasis markers were measured in the present work, and this interpretation therefore remains speculative.

Taken together, these data provide a mechanistic rationale for exploring VALP–DFO combination therapy, particularly in chronic epilepsy contexts characterized by elevated oxidative stress, iron dysregulation, or vulnerability to VALP-related toxicity.

This study has several limitations. First, no naïve or sham-operated (non-penicillin) control group was included; therefore, the relative contributions of penicillin-induced epileptiform activity versus surgical trauma, urethane anesthesia, or the intracortical injection procedure itself to the observed biochemical changes cannot be fully disentangled, particularly given reports that urethane anesthesia alone can modulate nitric oxide synthase expression independent of any pathological stimulus [33]. In addition, the assay used in this study measured nitrite/nitrate surrogate markers of nitric oxide metabolism rather than direct gaseous nitric oxide or nitric oxide synthase activity. Second, baseline spike amplitude differed significantly among groups prior to VALP/DFO administration, suggesting pre-existing inter-animal variability and/or transient handling, injection, or procedural effects. Percentage changes from baseline were used only descriptively and not as a statistical correction for baseline imbalance. Accordingly, the early post-treatment separation between groups should not be interpreted as a purely pharmacodynamic effect, and percentage change from baseline was used only for descriptive purposes. Third, this acute penicillin model reflects predominantly GABAergic disinhibition and does not capture chronic epileptogenesis, spontaneous seizures, or long-term network remodeling; therefore, extrapolation to chronic epilepsy or disease-modifying effects is not supported by the present data. Fourth, behavioral seizure severity, such as Racine scores, seizure latency and duration, survival, and cognitive outcomes, was not assessed. Therefore, the present study cannot determine whether the observed electrophysiological or biochemical changes translate into functional benefit or neuroprotection. Fifth, direct measurements of iron homeostasis, ferroptosis-related biomarkers such as GPX4, ACSL4, and SLC7A11, lipid peroxidation, mitochondrial function, and nitric oxide synthase activity were not performed. Accordingly, interpretations involving ferroptosis, iron-dependent mechanisms, mitochondrial pathways, or NOS signaling remain hypotheses for future testing rather than established mechanisms. Finally, only male rats were used. Therefore, potential sex-related differences and the influence of the estrous cycle on seizure susceptibility and oxidative stress responses were not evaluated.

## 5. Conclusions

Sodium valproate and deferoxamine each reduced spike frequency and amplitude and improved oxidative and nitrosative stress markers in this acute penicillin-induced model of epileptiform activity. The VALP + DFO combination did not provide additional anticonvulsant efficacy over VALP monotherapy, as spike frequency and spike amplitude were not further suppressed. The combination was associated with selected biochemical differences, particularly lower nitrite/nitrate surrogate levels and higher hippocampal TAS, but these findings should be regarded as secondary and exploratory given the acute model, limited sample size, and absence of a non-penicillin control group. These findings do not establish neuroprotection, disease modification, or an additive anticonvulsant effect of DFO. They provide only a hypothesis-generating rationale for further investigation of iron-dependent oxidative mechanisms and nitric oxide signaling in antiseizure therapy. Future studies using chronic epilepsy models, behavioral and cognitive outcomes, neuronal and histological assessments, direct measurements of iron homeostasis and ferroptosis-related biomarkers, and adequately powered designs are needed to determine whether VALP + DFO has functional or disease-modifying relevance.

## Figures and Tables

**Table 1:** Linear mixed-effects analysis of longitudinal spike frequency and amplitude.

Outcome	Effect	Test Statistic	df	*p* Value
Spike frequency	Group	χ^2^ = 309.71	6	<0.001
Spike frequency	Time	χ^2^ = 937.15	4	<0.001
Spike frequency	Group × time	χ^2^ = 161.96	3	<0.001
Spike amplitude	Group	χ^2^ = 341.78	6	<0.001
Spike amplitude	Time	χ^2^ = 856.73	4	<0.001
Spike amplitude	Group × time	χ^2^ = 226.75	3	<0.001

**Table 2:** Mean spike frequency values (spikes/min) recorded over 180 min following treatment (VALP, DFO, VALP + DFO, or saline) administration.

Groups	1st	30th	60th	90th	120th	150th	180th
Penicillin (*n* = 7)	115.20 ± 4.56	117.00 ± 4.84	112.00 ± 2.45	108.20 ± 2.62	103.30 ± 2.82	100.60 ± 2.74	98.70 ± 2.70
VALP (500 mg/kg, *n* = 7)	118.50 ± 3.74 *	81.30 ± 2.67 *	77.45 ± 3.45 *	74.50 ± 3.17 *	70.00 ± 3.72 *	60.00 ± 2.47 *	54.20 ± 2.74 *
DFO (100 mg/kg, *n* = 7)	116.00 ± 3.09	100.60 ± 3.11	94.30 ± 3.11	83.40 ± 2.94	72.00 ± 5.90 *	65.20 ± 3.89 *	62.00 ± 4.75 *
DFO (100 mg/kg) + VALP (500 mg/kg, *n* = 7)	113.00 ± 5.67 *	82.60 ± 5.74 *	78.60 ± 6.30 *	75.80 ± 5.68 *	69.80 ± 4.73 *	61.00 ± 5.48 *	58.00 ± 4.20 *

Values are expressed as mean ± SEM. * *p* < 0.0083 (Bonferroni-adjusted threshold for Mann–Whitney U comparisons following a significant Kruskal–Wallis test) compared with the penicillin group at the corresponding time point.

**Table 3:** Mean spike amplitude values (µV) recorded over 180 min following treatment (VALP, DFO, VALP + DFO, or saline) administration.

Groups	Baseline	1st	30th	60th	90th	120th	150th	180th
Penicillin (*n* = 7)	0.155 ± 0.04	0.163 ± 0.05	0.130 ± 0.03	0.117 ± 0.06	0.113 ± 0.04	0.091 ± 0.07	0.085 ± 0.04	0.079 ± 0.03
VALP 500 mg (*n* = 7)	0.025 ± 0.06	0.084 ± 0.03 *	0.058 ± 0.04 *	0.047 ± 0.05 *	0.044 ± 0.06 *	0.040 ± 0.05 *	0.038 ± 0.04 *	0.036 ± 0.04 *
DFO 100 mg/kg (*n* = 7)	0.028 ± 0.05	0.089 ± 0.05 *	0.071 ± 0.05 *	0.068 ± 0.03 *	0.062 ± 0.06 *	0.056 ± 0.05 *	0.050 ± 0.04 *	0.048 ± 0.06 *
DFO 100 mg/kg + VALP 500 mg (*n* = 7)	0.021 ± 0.03	0.092 ± 0.03 *	0.057 ± 0.04 *	0.045 ± 0.04 *	0.041 ± 0.05 *	0.025 ± 0.05 *	0.028 ± 0.06 *	0.026 ± 0.06 *

Values are expressed as mean ± SEM. * *p* < 0.0083 (Bonferroni-adjusted threshold for Mann–Whitney U comparisons following a significant Kruskal–Wallis test) compared with the penicillin group at the corresponding time point.

**Table 4:** Serum TAS, TOS, and OSI Levels in Experimental Groups.

Groups	TAS, mmol Trolox Eq/L	TOS, μmol H_2_O_2_ Eq/L	OSI, AU
Penicillin (*n* = 7)	0.67 ± 0.06	60.96 ± 0.6	9.02 ± 0.8
VALP 500 mg/kg (*n* = 7)	1.66 ± 0.07	24.5 ± 0.4	1.47 ± 0.6 ^+^
DFO 100 mg/kg (*n* = 7)	0.88 ± 0.05 *	17.40 ± 0.7	1.97 ± 0.5 ^#^
VALP 500 mg/kg + DFO 100 mg/kg (*n* = 7)	1.35 ± 0.04 **	26.86 ± 0.6 ***	1.98 ± 0.9 ^#^

Values expressed as mean ± SEM. The mean difference is significant at the 0.05 level. * *p* < 0.05; different compared to Penicillin, VALP and VALP + DFO groups. ** *p* < 0.01; different compared to Penicillin, VALP and DFO groups. *** *p* < 0.01; different compared to Penicillin and DFO groups. ^+^ *p* < 0.05; different compared to Penicillin, DFO and VALP + DFO groups. ^#^ *p* < 0.001; different compared to Penicillin group.

**Table 5:** Hippocampal Tissue TAS, TOS, and OSI Levels in Experimental Groups.

Groups	TAS, mmol Trolox/mg Protein	TOS, μmol H_2_O_2_/mg Protein	OSI, AU
Penicillin (*n* = 7)	2.27 ± 0.08	245.80 ± 3.5	11.52 ± 0.6 ***
VALP 500 mg/kg (*n* = 7)	2.64 ± 0.09	168.11 ± 4.4 **	6.92 ± 0.2
DFO 100 mg/kg (*n* = 7)	1.90 ± 0.07	167.96 ± 6.3 **	9.22 ± 0.4
VALP 500 mg/kg + DFO 100 mg/kg (*n* = 7)	3.67 ± 0.08 *	181.41 ± 4.2 **	5.16 ± 0.2 ^#^

Values expressed as mean ± SEM. The mean difference is significant at the 0.05 level. * *p* < 0.01; significantly different compared with Penicillin and DFO groups. ** *p* < 0.001; significantly different compared with Penicillin group. *** *p* < 0.01; significantly different compared with VALP and VALP + DFO groups. ^#^ *p* < 0.05; significantly different compared with Penicillin and DFO groups.

## Data Availability

The data presented in this study are available from the corresponding author upon reasonable request.

## Abbreviations

The following abbreviations are used in this manuscript:

DFO	Deferoxamine
ECoG	Electrocorticography
ELISA	Enzyme-linked immunosorbent assay
i.c.	Intracortical
i.p.	Intraperitoneal
nNOS	Neuronal nitric oxide synthase
NO	Nitric oxide
OSI	Oxidative stress index
PTZ	Pentylenetetrazole
RNS	Reactive nitrogen species
ROS	Reactive oxygen species
TAS	Total antioxidant status
TOS	Total oxidant status
VALP	Valproate
